# Structural Evolution and Cascading Propagation of Supply Risk in the Global Nickel Industry Chain: A Multilayer Network Approach

**DOI:** 10.3390/e28080937

**Published:** 2026-08-21

**Authors:** Yi Liang, Xiaoduo Wang, Han Liu, Hao Wang

**Affiliations:** 1 School of Management, Hebei GEO University, Shijiazhuang 052161, China; louisliang@hgu.edu.cn (Y.L.); 250701120200002@hgu.edu.cn (X.W.); 18190130811@163.com (H.L.); 2 Strategy and Management Base of Mineral Resources in Hebei Province, Hebei GEO University, Shijiazhuang 052161, China; 3 School of Business, Hebei University of Engineering Science, Shijiazhuang 050020, China

**Keywords:** nickel industry chain, complex networks, supply risk, multilayer networks, cascading-failure model

## Abstract

Geopolitical conflicts, resource-protection policies, and unexpected disruptions have heightened supply-security concerns across the global nickel industry chain. This study constructs a multilayer trade network based on complex network theory to characterize structural evolution across the upstream, midstream, and downstream segments and applies a cascading-failure model to simulate the propagation of supply risks. There are four main findings: (1) The global nickel trade network exhibits pronounced layer heterogeneity, with the midstream layer acting as the principal amplifier of cascading failure risks. (2) Nodes with high centrality and broad cross-layer participation largely coincide with the countries that generate the largest systemic risks. (3) A small group of countries controls most trade flows and dominates risk transmission. (4) The center of systemic risk is shifting from traditional industrial and trading economies toward resource suppliers and countries that integrate resource extraction with processing. These findings support a risk-governance strategy based on diversified supply sources, dynamic monitoring of critical nodes, improved resilience in midstream smelting and refining, strategic resource stockpiling, and the circular utilization of nickel resources.

## 1. Introduction

Driven by the global energy transition and the upgrading of advanced manufacturing, nickel has become a strategically important mineral. Beyond its established uses in specialty alloys and electroplating, nickel is a key input for electric-vehicle batteries and energy-storage systems and has consequently become central to national critical-mineral strategies. Demand continues to increase as economies pursue carbon-neutrality targets, while the balance between supply and demand remains structurally tight. At the same time, nickel resources are distributed highly unevenly. Laterite deposits are concentrated in equatorial countries such as Indonesia and the Philippines, whereas sulfide deposits occur mainly at higher latitudes, including Australia and Canada. This spatial mismatch between supply and consumption, compounded by geopolitical conflicts, frequent adjustments to resource-export policies and increasingly stringent environmental constraints, has placed the global nickel trade network under sustained pressure. Local supply disruptions can readily propagate across regions and production stages through trade links, generating systemic risks throughout the industry chain. Identifying the structural characteristics of the network and the principal nodes of risk transmission is therefore essential for mitigating supply-chain disruptions.

Critical minerals have increasingly attracted attention in resource economics, international trade and supply-chain security. Existing research on critical-mineral trade has broadly followed two approaches. The first adopts a national or regional perspective and examines the roles of individual countries, their dependence on imports and their exposure to supply risks. Indicators such as the composition of import sources, trade concentration and external dependence are commonly used to assess critical-mineral supply risks and their implications for industrial security [1,2]. The second approach takes a global trade-network perspective, representing countries as nodes and trade relationships as edges. This framework has been applied to lithium [3], aluminum [4], scrap copper [5] and other critical minerals to examine structural evolution, identify core countries and determine the factors shaping network formation. Such analyses typically combine complex-network metrics with exponential random graph models (ERGMs) [6] or temporal exponential random graph models (TERGMs) [7,8] to assess the effects of resource endowment, economic size, geographical distance, institutional conditions and geopolitical relationships on trade-network structure. However, most existing network analyses focus on a single mineral product or an isolated segment of an industry chain. They commonly construct single-layer networks for ores, refined materials or final products, or select a limited number of representative commodities [9,10]. Although these approaches reveal the trade patterns of specific products or production stages, they cannot fully represent the connections linking resource extraction, smelting and refining, and downstream manufacturing. Nor can they adequately capture the extent to which countries are embedded across multiple stages. In the nickel industry, upstream ore supply, midstream smelting and processing, and downstream product trade differ markedly in their participants, trade routes, network functions and exposure to supply risks. A single-layer network is therefore insufficient to reveal the resulting layer heterogeneity and cross-layer interactions. An integrated multilayer network encompassing upstream resources, midstream processing and downstream products is needed to characterize the structural evolution of the global nickel trade system.

Resilience theory has also been incorporated into analyses of critical-mineral trade networks, particularly for strategic minerals such as lithium, cobalt and nickel. Two main lines of research have emerged. The first examines structural robustness under short-term external shocks. Random attacks, targeted attacks, and node or edge failures are simulated to evaluate changes in network connectivity, efficiency and functional performance, thereby measuring the ability of a trade network to withstand and recover from sudden disruptions [11,12]. The second uses long-term trade data to evaluate how resilience evolves over time. Indicators of network concentration, hierarchy, assortativity and connectivity are incorporated into resilience-assessment frameworks and compared across years [13,14]. Other studies have investigated the effects of geopolitical conflict, export restrictions, natural disasters, public-health emergencies and financial-market volatility, showing that trade dependence, concentrated supply and market expectations can amplify the effects of external shocks [15,16]. This literature provides an important foundation for understanding critical-mineral supply risks, but existing assessments rely predominantly on static structural indicators or aggregate measures of network performance. At the micro level, supplier selection under uncertainty is itself a multi-criteria decision problem, which complements network-level resilience analysis by linking structural vulnerability to actual sourcing decisions [17]. They therefore describe resilience mainly as an observed outcome, while offering less insight into how disruptions propagate between production stages, whether particular layers amplify risk, and which countries can initiate large cascading failures. Multilayer network methods offer a means of addressing these limitations. They have been applied to cascading-failure vulnerability in intermodal transportation networks and resilience assessment in interconnected urban infrastructure systems [18,19], but their use in analyzing the structure and risk-transmission processes of critical-mineral industry chains remains limited [20,21]. Risk in a critical-mineral trade network is not determined solely by the structural importance of an individual node. Trade volume, cross-layer connections, position within the industry chain and node substitutability jointly shape both the likelihood and the consequences of propagation [22,23,24,25]. A highly central country may facilitate trade under normal conditions while also providing an efficient pathway for shock transmission. Conversely, a country with relatively few connections may still generate substantial systemic effects if it supplies a large and difficult-to-replace share of a critical input. Combining multilayer structural analysis with cascading-failure simulation can therefore reveal how risk moves across production stages, where it is amplified and which nodes are most capable of transmitting it throughout the network.

Specifically, although previous studies have investigated multilayer critical-mineral trade networks, nickel-industry-chain risks, and cascading propagation, clear differences remain in network structure, interlayer coupling mechanisms, and risk-propagation rules. Existing literature on critical-mineral trade networks commonly defines countries as nodes and trade relationships as edges, with an emphasis on network evolution, core countries, and formation mechanisms. These studies help reveal global trade patterns and nodal positions; however, most focus on a single mineral product or a specific segment of the industry chain. They therefore provide limited insights into the cross-layer connections among upstream resource supply, midstream smelting and refining, and downstream product trade. Prior research on nickel-related industry-chain risks has mainly examined trade patterns, import dependence, resource security, and supply-risk assessment. Although this research reveals trade concentration and national exposure to nickel-supply risks, it provides little explanation of how risks propagate across different stages of the industry chain, which layers amplify shocks, and which countries can trigger cascading failures.

Meanwhile, multilayer networks and cascading-failure models have been adopted to analyze risk propagation in critical-mineral systems and supply chains, providing an important methodological basis for this study. Nevertheless, this body of work still differs from the present study in terms of interlayer coupling, cascading rules, and risk metrics. Existing research has generally focused on multi-product networks, supply-chain-layer associations, or changes in overall network function after node failures. By contrast, this study divides the global nickel industry chain into upstream resource, midstream smelting, and downstream product layers, and constructs a directed and weighted multilayer trade network. In terms of interlayer coupling, interlayer links are established between replica nodes of the same country in adjacent industry-chain layers, and interlayer loss transmission is characterized according to nickel material-conversion relationships. In terms of risk identification, this study combines node activity, the multilayer participation coefficient, centrality metrics, avalanche size, avalanche ratio, and representative propagation pathways to reveal the relationships among cross-layer embeddedness, layer-specific structural differences, and cascading supply-risk diffusion. Thus, this study does not simply replicate existing multilayer-network or cascading-propagation research; instead, it extends prior work by incorporating complete nickel-industry-chain stratification, explicit interlayer coupling, and dynamic risk-propagation simulation.

Overall, existing studies provide an important foundation for understanding critical-mineral trade patterns, nickel-industry-chain risks, and supply-chain resilience, but several research gaps remain. First, in terms of research scope, most existing studies focus on individual countries, single products, or specific segments of the industry chain, and therefore do not fully characterize the multilayer trade structure connecting upstream resources, midstream smelting, and downstream products in the global nickel industry chain. Second, in terms of interlayer coupling, few studies explicitly incorporate input–output linkages between adjacent industry-chain stages for the same country, which hinders explanations of how cross-layer national embeddedness affects risk propagation. Third, in terms of propagation mechanisms, existing resilience studies largely rely on static metrics, trade-dependence measures, or localized attack simulations, and pay insufficient attention to how supply shocks diffuse iteratively through intralayer trade relationships and interlayer industry-chain linkages. To fill these research gaps, this study constructs a multilayer trade network for the global nickel industry chain, introduces node activity and the multilayer participation coefficient to characterize national embeddedness and cross-layer connectivity, and applies a cascading-failure model to simulate the propagation of supply shocks across the multilayer network. On this basis, the framework is used to identify critical risk-source countries, layer-specific propagation characteristics, and representative propagation pathways.

Compared with previous studies, this study makes three main contributions. First, in terms of network structure and data coverage, it uses global trade data for nickel-related products from 2012 to 2024 and divides the nickel industry chain into upstream resource, midstream smelting, and downstream product layers. It thereby constructs a directed and weighted multilayer trade network, extending trade-network research beyond single products or isolated industry-chain segments. Second, in terms of interlayer coupling and structural metrics, this study establishes interlayer connections between replica nodes of the same country in adjacent industry-chain layers, and introduces node activity and the multilayer participation coefficient to measure countries’ cross-layer embeddedness and potential risk-transmission capacity. Third, in terms of propagation mechanisms and empirical identification, this study integrates multilayer structural analysis with cascading-failure simulation to examine how supply risks spread across upstream, midstream, and downstream layers under different shock intensities. It identifies critical risk-source countries, layer-specific amplification effects, and representative propagation pathways, thereby providing evidence for supply-risk monitoring and resilience governance in the global nickel industry chain.

## 2. Materials and Methods

### 2.1. Data Sources

The analysis covered nickel ore in the upstream layer, five product categories in the midstream layer, and fifteen product categories in the downstream layer. Import and export trade records were obtained from the United Nations Comtrade Database, and trade flows involving less than 5 kg were removed. The study period spanned 2012–2024, with 2020 and 2024 selected for detailed comparison because they represented distinct supply-disruption conditions and the latest network topology available in the dataset. To ensure comparability across stages, trade was measured in nickel-content equivalents: the trade volume at each stage equaled the total mass of contained nickel across all products assigned to that stage [26]. Table 1 summarizes the product coverage and conversion coefficients after preprocessing.

### 2.2. Methods

#### 2.2.1. Construction of the Multilayer Network

A directed, weighted multilayer network was constructed for the upstream, midstream, and downstream segments of the nickel industry chain. This network is formalized by the following supra-adjacency matrix:

(1) $$G=\left[\begin{array}{ccc} A^{\left[1\right]} & O^{\left[1,2\right]} & 0\\ 0 & A^{\left[2\right]} & O^{\left[2,3\right]}\\ 0 & 0 & A^{\left[3\right]} \end{array}\right]$$

where $A^{\left[1\right]}$, $A^{\left[2\right]}$, and $A^{\left[3\right]}$ denote the intra-layer adjacency matrices for the upstream, middle, and downstream sections, respectively. $\omega_{ij}^{\left[\alpha \right]}$ is an element of $A^{\left[\alpha \right]}$. For layer α, if nodes i and j are connected, $\omega_{ij}^{\left[\alpha \right]}$ denotes the corresponding edge weight; otherwise, $\omega_{ij}^{\left[\alpha \right]}=0$. $O^{\left[1,2\right]}$ and $O^{\left[2,3\right]}$ denote the interlayer coupling matrices between the upstream and midstream sections, as well as between the midstream and downstream sections. The inter-layer edge represents the input–output relationship between copy nodes of the same country across adjacent industrial chain layers. Specifically, $O^{\left[1,2\right]}$ connects only upstream nodes with midstream nodes within the same country, while $O^{\left[2,3\right]}$ connects only midstream nodes with downstream nodes within the same country. If no corresponding node exists for a given country at a neighboring layer, no inter-layer edge is established, and its corresponding inter-layer weight is set to 0. Therefore, the interlayer adjacency matrices are defined as follows:

(2) $$O_{ij}^{\left[1,2\right]}=\left\{\begin{array}{c} \lambda_{12},i=j,\;i\in V^{\left[1\right]}\cap V^{\left[2\right]}\\ 0,\;otherwise \end{array}\right.$$

(3) $$O_{ij}^{\left[2,3\right]}=\left\{\begin{array}{c} \lambda_{23},i=j,\;i\in V^{\left[2\right]}\cap V^{\left[3\right]}\\ 0,\;otherwise \end{array}\right.$$

The interlayer weights are determined by the conversion relationships of nickel materials between adjacent stages of the industry chain. Considering that material losses occur during ore supply, smelting and processing, and product manufacturing, and based on the nickel recovery-rate ranges and material-flow relationships reported in the relevant literature [27,28], the conversion coefficient from the upstream resource stage to the midstream smelting and processing stage is set as $\lambda_{12}=0.95$, while the conversion coefficient from the midstream smelting and processing stage to the downstream product stage is set as $\lambda_{23}=0.90$. These values are used as the baseline interlayer conversion coefficients.

#### 2.2.2. Layer-Specific Network Topology

Network topological metrics quantify the structural configuration, connectivity efficiency, trade intensity, and latent supply risks of the nickel trade network from two perspectives: the network as a whole and individual nodes. Accordingly, global network metrics and nodal metrics were used to analyze the upstream, midstream, and downstream layers.

(1) Global network metrics. Average degree, average weighted degree, average path length, and average clustering coefficient were used to characterize the overall network structure [29]. Average degree measures the mean number of trade connections between network nodes and therefore reflects network density. Average weighted degree integrates trade-volume weights to quantify the overall trade-engagement intensity of each economy.

Average path length measures the mean number of steps required to connect pairs of trading countries. A smaller value indicates more efficient resource circulation through the network. Average path length is defined as follows:

(4) $$L=\frac{1}{n(n-1)}\sum_{i,j=1,i\neq j}^{n}d_{ij}$$

where L is the average path length of the network, and $d_{ij}$ is the shortest-path distance between nodes i and j.

The average clustering coefficient measures local node agglomeration and captures the tendency of neighboring trading countries to form interconnected groups. It is defined as follows:

(5) $$C=\frac{1}{n}\sum_{i=1}^{n}\frac{e_{i}}{m_{i}(m_{i}-1)}$$

where C is the average clustering coefficient of the network; $m_{i}$ is the number of neighbors of node i; and $e_{i}$ is the number of links among the neighbors of node i.

(2) Nodal metrics. Strength centrality, betweenness centrality, and PageRank centrality were used to identify the structural position of each trade node [30]. Among them, total strength is adopted for strength centrality, which reflects the scale of nodes’ participation in trade and the intensity of trade connections. Betweenness centrality is calculated based on the directed topological shortest path. The length of each existing trade edge is set to 1, and unreachable node pairs are excluded from the average path-length calculation. It is normalized by (N−1)(N−2) to reflect the bridging role of nodes in the trade network and their control over trade paths. PageRank centrality combines the features of itself and adjacent nodes to comprehensively evaluate the core influence of economies in the nickel trade network. Its damping coefficient is set to 0.85, the convergence threshold is set to 0.001, and the sum of its scores equals 1.

#### 2.2.3. Multilayer Network Measures

To characterize cross-layer trade participation across the nickel industry chain, this study introduced two multilayer metrics. Unlike conventional single-layer measures, multilayer metrics capture the coupling of trade relationships across production stages and provide a more comprehensive assessment of each economy’s embeddedness and overall trade position. Node activity and the multilayer participation coefficient were used in the analysis.

(1) Node activity quantifies the number of layers in which a node maintains active trade ties and ranges from 0−M. It therefore measures the number of industry-chain stages in which an economy participates [31]. Because the network comprises three industrial layers (upstream, midstream, and downstream), node activity ranges from 0 to 3.
(2) Multilayer participation coefficient. The multilayer participation coefficient [30] describes how evenly a node’s trade links are distributed across distinct network layers and therefore captures differences in national participation across the nickel industry chain. It is defined as follows:

If node i has multiple edges in many layers, whereas node j has edges in only a few layers, node i has greater potential influence on the multilayer network. The multilayer participation coefficient $P_{i}$ captures the distribution of a node’s links across layers.

(6) $$P_{i}=\frac{M}{M-1}\left[1-\sum_{\alpha =1}^{M}{\left(\frac{k_{i}^{\left[\alpha \right]}}{o_{i}}\right)}^{2}\right],P_{i}\in [0,1]$$

where $k_{i}^{\left[\alpha \right]}$ denotes the degree in layer α of node i; $k_{i}^{\left[\alpha \right]}=\sum_{j\neq i}a_{ij}^{\left[\alpha \right]}$ and $o_{i}$ denotes the total degree of node i across all layers, $o_{i}=\sum_{\alpha }k_{i}^{\left[\alpha \right]}$.

#### 2.2.4. Multilayer Supply-Risk Propagation Model

The propagation of nickel supply risk can theoretically be regarded as a cascading failure or avalanche process on a weighted, directed, multilayer trade network. To describe this process formally, we consider a global nickel industry-chain network consisting of three layers: the upstream resource layer (ℓ=1), the midstream smelting and processing layer (ℓ=2), and the downstream product layer (ℓ=3). Let $\omega_{ij}^{\left[\ell \right]}$ denote the weight of the directed trade edge from country i to country j in layer ℓ. Let $x_{i}^{\left[\ell \right]}(t)$ denote the state of node i in layer ℓ at round t, where $x_{i}^{\left[\ell \right]}(t)=0$ indicates that the node is in a normal state and $x_{i}^{\left[\ell \right]}(t)=1$ indicates that it is in an infected state. Let $L_{i}^{\left[\ell \right]}(t)$ denote the cumulative supply loss experienced by country i in layer ℓ up to round t, and let $T_{i}^{\left[\ell \right]}$ denote the risk-bearing threshold of the corresponding node. The parameter α denotes the supply-shock intensity coefficient, while β denotes the infection-threshold adjustment coefficient [32]. A node becomes infected only when its cumulative loss exceeds $\beta T_{i}^{\left[\ell \right]}$. To capture the overall effect of a supply shock relative to a node’s risk-bearing capacity, we further define the effective shock-to-threshold ratio as κ=α/β. In the baseline simulations, β is fixed at 1, and the cascading propagation of risk across the nickel industry chain is examined by varying κ to represent different supply-shock intensities. The interlayer conversion coefficients from the upstream to the midstream layer and from the midstream to the downstream layer are denoted by $\lambda_{12}$ and $\lambda_{23}$, respectively.

When country s in layer ℓ is designated as the initial risk source, the model propagates the shock according to the bilateral trade weights between the source country and its importing countries. If country s exports nickel-related products to country j, the initial loss incurred by country j in the same layer is given by $\alpha \omega_{sj}^{\left[\ell \right]}$.

(1) Loss-update equations

At propagation round t, let $F^{\left[\ell \right]}(t)$ denote the set of newly infected nodes in layer ℓ. These newly infected nodes become risk sources in the subsequent round and transmit losses to importing countries through their outgoing trade links. The additional intralayer loss incurred by country j in layer ℓ is expressed as

(7) $$\Delta L_{j,intra}^{\left[\ell \right]}(t)=\alpha \sum_{i\epsilon F^{\left[\ell \right]}(t)}\omega_{ij}^{\left[\ell \right]}$$

This equation indicates that when country j imports nickel-related products from multiple newly infected suppliers, the resulting losses are aggregated across all affected suppliers, thereby capturing the cumulative effect of multiple supply shocks.

In addition to intralayer propagation, risk is transmitted across layers along the direction of the nickel industry chain. If country i experiences a cumulative loss in the upstream layer and has a corresponding replica node in the midstream layer, its upstream cumulative loss is transmitted to the midstream replica node according to the conversion coefficient $\lambda_{12}$. Similarly, if country i experiences a cumulative loss in the midstream layer and has a corresponding replica node in the downstream layer, its midstream cumulative loss is transmitted to the downstream replica node according to the conversion coefficient $\lambda_{23}$. The additional interlayer losses are given by

(8) $$\Delta L_{i,cross}^{\left[2\right]}(t)=\lambda_{12}L_{i}^{\left[1\right]}(t)$$

(9) $$\Delta L_{i,cross}^{\left[3\right]}(t)=\lambda_{23}L_{i}^{\left[2\right]}(t)$$

If a country does not have a corresponding node in an adjacent layer, no interlayer transmission occurs. Accordingly, the cumulative loss of a node in each round is determined jointly by its cumulative loss in the previous round, the additional intralayer loss, and the additional interlayer loss:

(10) $$L_{i}^{\left[\ell \right]}(t+1)=L_{i}^{\left[\ell \right]}(t)+\Delta L_{i,intra}^{\left[\ell \right]}(t)+\Delta L_{i,cross}^{\left[\ell \right]}(t)$$

Because the upstream layer has no preceding layer, its interlayer loss is set to zero: $\Delta L_{i,cross}^{\left[1\right]}(t)=0$.

(2) State-transition equations

When the cumulative loss of a node exceeds its infection threshold, the node transitions from the normal state to the infected state. Specifically, if $L_{j}^{\left[\ell \right]}(t+1)>\beta T_{j}^{\left[\ell \right]}$, then $x_{j}^{\left[\ell \right]}(t+1)=1$. Once infected, a node remains in the infected state in all subsequent rounds and does not return to the normal state.

**Figure 1:**
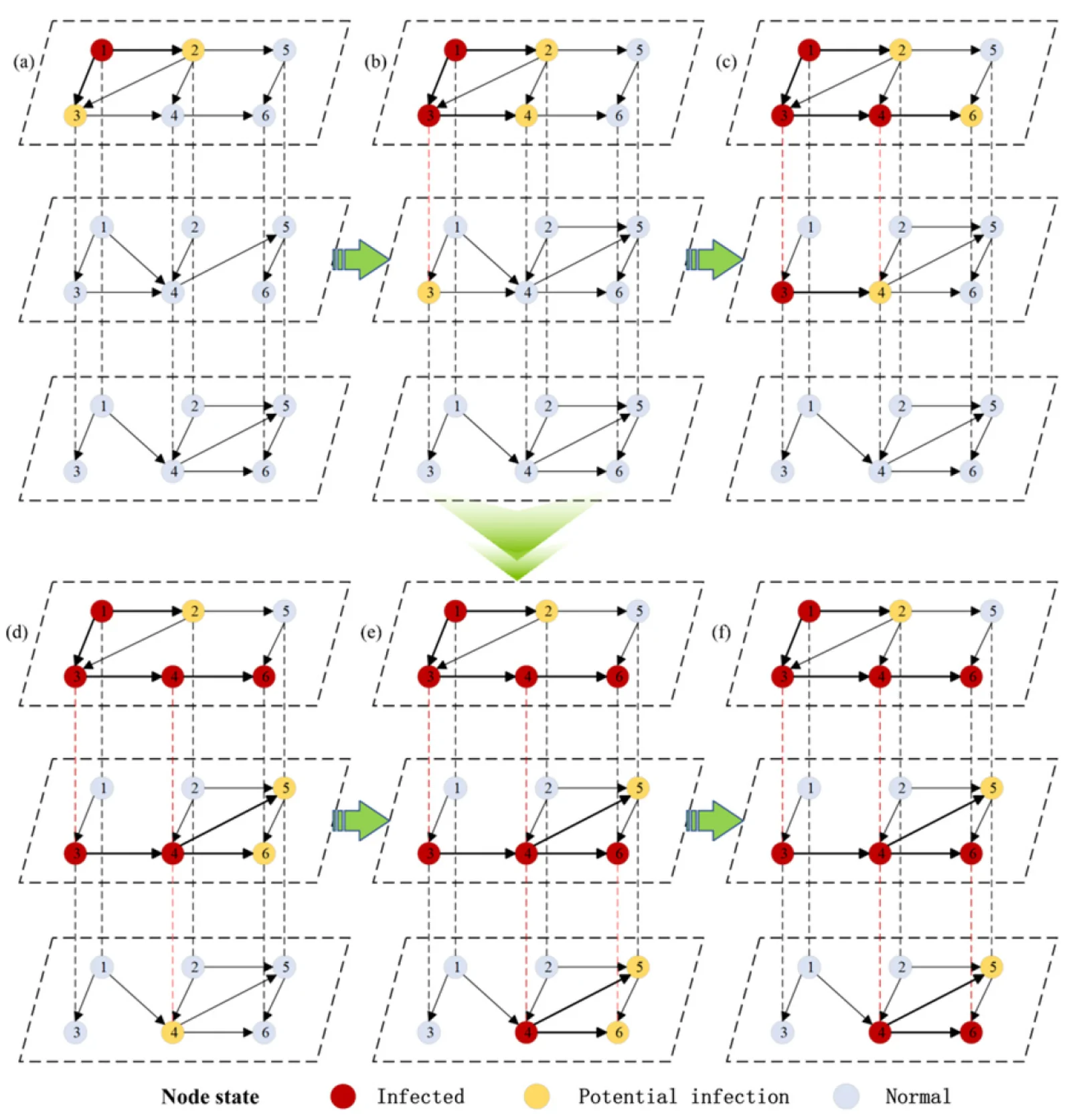
Propagation rules in the multilayer nickel industry-chain network. Note: (**a**) Initial state, with country 1 as the supply shock source; (**b**) shock propagation from country 1 to country 3 in the first layer; (**c**) country 3 becomes infected and the shock spreads to country 4; (**d**) country 4 is infected in the third layer; (**e**) continued diffusion in the multilayer network; (**f**) final propagation state. Numbers 1–6 denote countries.

The model adopts a synchronous updating mechanism. In each round, the additional losses of all nodes are first calculated simultaneously based on the currently newly infected nodes. The states of all nodes are then updated simultaneously according to their cumulative losses. In other words, the infection of an individual node does not immediately trigger another propagation event within the same round. The cascading process terminates when no new infected nodes emerge in a given round. The detailed propagation process of supply risk in the multilayer nickel industry-chain network is illustrated in Figure 1.

(3) Propagation-process pseudocode

Input: Three trade-weight matrices $W^{\left[1\right]}$, $W^{\left[2\right]}$, and $W^{\left[3\right]}$; interlayer mapping relationships; conversion coefficients $\lambda_{12}$ and $\lambda_{23}$; the effective shock-to-threshold ratio κ; infection-threshold and baseline risk-bearing thresholds $T_{i}^{\left[\ell \right]}$.

Output: Avalanche size and avalanche ratio.

① Initialize all country–layer nodes as normal, with $x_{i}^{\left[\ell \right]}(0)=0$ and $L_{i}^{\left[\ell \right]}(0)=0$.

② Select one country–layer node as the initial risk source, set its state to infected, and add it to the round-0 propagation set.

③ For each newly infected node in the current round, calculate the additional intralayer loss incurred by its importing countries through its outgoing trade edges. The loss equals α multiplied by the corresponding trade weight.

④ If the currently infected node has a replica node representing the same country in the adjacent downstream layer, calculate the additional interlayer loss using the corresponding interlayer conversion coefficient.

⑤ Aggregate the losses transmitted by all failed suppliers and through interlayer propagation, and update the cumulative loss of each node.

⑥ Examine all nodes that have not yet been infected. If a node’s cumulative loss exceeds $\beta T_{i}^{\left[\ell \right]}$, mark it as newly infected in the next round.

⑦ Use the newly infected nodes from the current round as propagation sources in the next round, and repeat Steps 3–6.

⑧ Terminate the simulation when no new infected nodes emerge in the network.

⑨ Finally, count all infected nodes to obtain the avalanche size, and calculate the ratio of the avalanche size to the total number of country–layer nodes to obtain the avalanche ratio.

To quantify the extent of the impact caused by an initial supply shock, the total number of country–layer nodes that are infected by the end of the propagation process is defined as the avalanche size. For an initial risk source s, the total avalanche size is given by

(11) $$A_{s}=\sum_{\ell =1}^{3}\sum_{i}x_{i}^{\left[\ell \right]}(t_{end})$$

The avalanche ratio [33] is defined as the avalanche size divided by the total number of country–layer nodes in the network:

(12) $$R_{s}=\frac{A_{s}}{N^{\left[1\right]}+N^{\left[2\right]}+N^{\left[3\right]}}$$

where $N^{\left[\ell \right]}$ denotes the number of nodes in layer ℓ. The avalanche size and avalanche ratio are also calculated separately for the upstream, midstream, and downstream layers to identify differences in risk propagation and amplification across the three stages of the industry chain. A larger avalanche size and a higher avalanche ratio indicate that the initial risk source exerts a greater influence on risk propagation throughout the nickel industry chain. Assuming that each country–layer node may act as an initial risk source, we simulate the propagation of supply-chain risk under the effective shock-to-threshold ratio κ of 1, 1.5, 2, 2.5, 3, 3.5, 4, 4.5, 5, 6, 7, 10. The simulation code was implemented using MATLAB R2025b.

## 3. Results

Global and nodal metrics were first used to describe the topology of the upstream, midstream, and downstream trade networks. Multilayer-specific measures were then introduced to characterize cross-layer structure. Finally, the cascading-failure model was used to identify critical risk-source countries, compare propagation across layers, and trace the magnitude and pathways of risks originating from representative countries.

### 3.1. Topological Characteristics of the Nickel Trade Network

#### 3.1.1. Network-Level Characteristics

**Figure 2:**
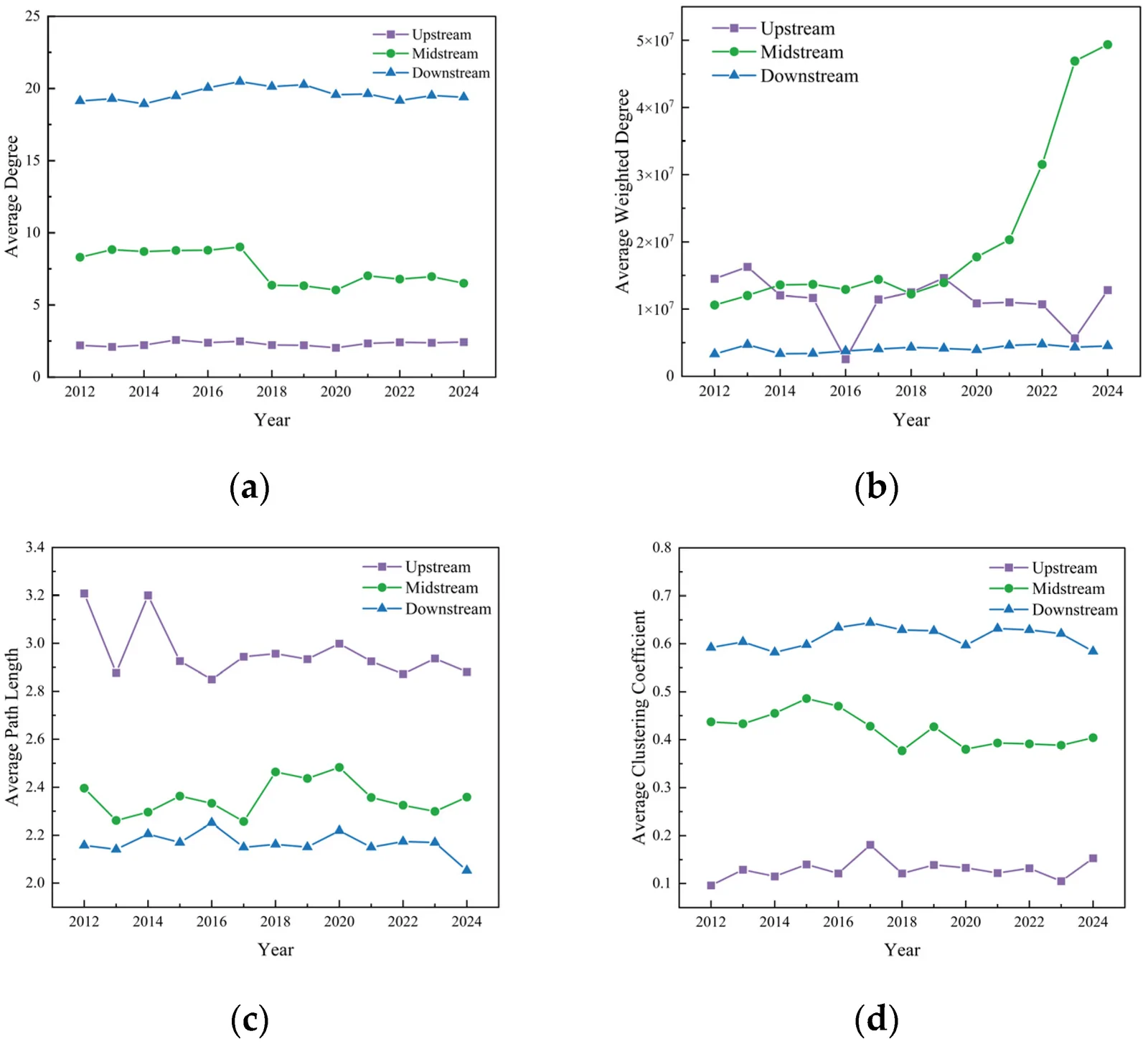
Network-level indicators for the upstream, midstream, and downstream nickel networks, 2012–2024. Note: (**a**–**d**) respectively represent the average degree, average weighted degree, average path length, and average clustering coefficient for each year.

A summary of key indicators reflecting the overall characteristics of the upstream, midstream, and downstream trade network from 2012 to 2024, aimed at examining changes in its network topology structure (Figure 2).

From the perspective of overall evolution, the multi-layer network of global nickel trade exhibits significant hierarchical heterogeneity. The downstream network consistently maintains high average degree and average clustering coefficient, alongside a low average path length, which reflects that downstream product trade features wide coverage, a high degree of local agglomeration and strong accessibility among countries. Driven by growing demand from stainless steel, power batteries and related manufacturing industries, trade linkages for downstream products keep expanding, and cross-border connections in end-manufacturing and consumption segments are further strengthened.

The upstream network displayed the opposite pattern. Its average degree and clustering coefficient were relatively small, whereas its average path length was relatively large, indicating that trade in primary nickel resources was strongly constrained by resource endowments and organized through a sparse network. Because nickel ores are concentrated in a small number of resource-rich countries, upstream trade offered limited substitutability and redundancy and was particularly exposed to resource policies and geopolitical conditions. Indonesia completely banned nickel-ore exports in 2020 and promoted domestic smelting and downstream processing, further altering cross-border ore flows and limiting the emergence of an open and balanced upstream network. The midstream network changed most markedly. Its average degree declined around 2018 and remained below earlier levels, whereas its average weighted degree rose rapidly after 2020 and exceeded those of the other layers. The midstream layer therefore shifted from growth in the number of connections toward concentration in trade intensity. This transition was associated with Indonesia’s expansion of domestic smelting, investment in ferronickel, nickel matte, and battery-material capacity, and the deep involvement of Chinese firms in Indonesian processing. By 2024, global nickel demand had continued to rise, while newly added supply was highly concentrated in Indonesia. The midstream network consequently displayed node consolidation and flow concentration.

The average clustering coefficient of the midstream network was initially high but subsequently declined to a moderate level, while average path length increased around 2018–2020. These changes indicate not a general loss of connectivity but a transition from decentralized connections to hub-centered organization. Routing larger trade volumes through a small number of processing and refining centers creates economies of scale but also increases the likelihood that disturbances at core nodes will propagate downstream.

Volatility in the international nickel market in 2022 highlighted this structural vulnerability. Following the escalation of the Russia–Ukraine conflict, uncertainty surrounding Russian nickel supply increased, and the London Metal Exchange subsequently suspended nickel trading. These events demonstrated the sensitivity of the global market to tight supply, concentrated positions, and fragile liquidity. Although the episode was expressed primarily through sharp price movements and financial-market volatility, it also exposed systemic risks arising from concentrated production of nickel intermediate products and refined nickel. In 2024, social unrest in New Caledonia, delays in Indonesian mining quotas, and restrictions on Russian nickel briefly intensified market concerns. Nevertheless, nickel prices remained under pressure from continued Indonesian capacity expansion and expectations of oversupply. This pattern is consistent with the rise in midstream average weighted degree without a corresponding recovery in average degree: recent network growth has amplified core channels rather than broadly dispersing participation.

Taken together, the four topological indicators demonstrate that the global nickel trade network did not merely expand in scale. Instead, it underwent a hierarchical restructuring driven by concentrated upstream resource supply, centralized midstream processing capacity, and dispersed downstream demand. The downstream layer exhibited broad market coverage and comparatively strong network resilience; the upstream layer remained highly resource-dependent; and the midstream layer experienced the greatest structural change and the strongest accumulation and amplification of supply risks. The central governance question is therefore not only whether total supply is sufficient, but also whether excessive concentration in a few processing centers amplifies cross-layer risk transmission.

#### 3.1.2. Node-Level Characteristics

Rank the centrality of trade networks across the upstream, midstream, and downstream segments of the nickel resource industry chain and examine their evolutionary patterns (Table 2). Overall, China retained a pronounced advantage in strength centrality, while resource suppliers, processing economies, and established trade hubs performed distinct structural roles across the three layers.

(1) Strength centrality. China remained the dominant nodal core of the nickel trade network, although the leading economies differed across layers. In the upstream network, the Philippines ranked first in 2024 and Indonesia entered the top three, indicating that trade intensity at the ore-supply stage had become concentrated in resource-rich countries. New Caledonia and South Korea remained in the top five, confirming the continuing importance of established suppliers and major importing processors. China ranked first in the midstream layer in both years, Indonesia remained second, and Japan rose in the ranking, showing that smelting, refining, and material processing were concentrated in a few economies with substantial processing capacity. China also ranked first downstream, while Indonesia rose and Germany, the United States, and India entered the top five. Downstream trade was therefore supported by both established manufacturing economies and expanding demand in emerging industrial markets.
(2) Betweenness centrality. Changes in major bridging nodes were more pronounced. In the upstream network, China rose in rank, while the United States, the Netherlands, Canada, and South Africa entered the top five, indicating that transit and allocation functions had become concentrated in major consuming economies, trade hubs, and resource-related countries. China, Germany, and Belgium ranked highly in the midstream network and therefore played major bridging roles along processing trade routes. In the downstream network, Germany and the United States occupied the top positions in 2024, China ranked third, and India and France entered the top five. Intermediation in downstream trade thus reflected the combined effects of manufacturing capacity, consumer demand, and regional distribution networks.
(3) PageRank centrality. Influence within the nickel trade network also differed by layer. In 2024, the Netherlands ranked first upstream, China and Canada remained highly ranked, and Brazil entered the top five. Upstream influence therefore depended not only on resource-supply volume but also on re-export trade, market connectivity, and regional allocation capacity. China remained first in the midstream layer, followed by major European processing and trading economies, including Germany, the Netherlands, Belgium, and Italy. Downstream, Germany replaced the United Kingdom in first place, while the Netherlands, India, Spain, and France entered the top five. Influence in downstream trade was thus concentrated in European manufacturing and trade centers, with emerging economies such as India becoming more important.

#### 3.1.3. Multi-Layer Network Metric Analysis

**Figure 3:**
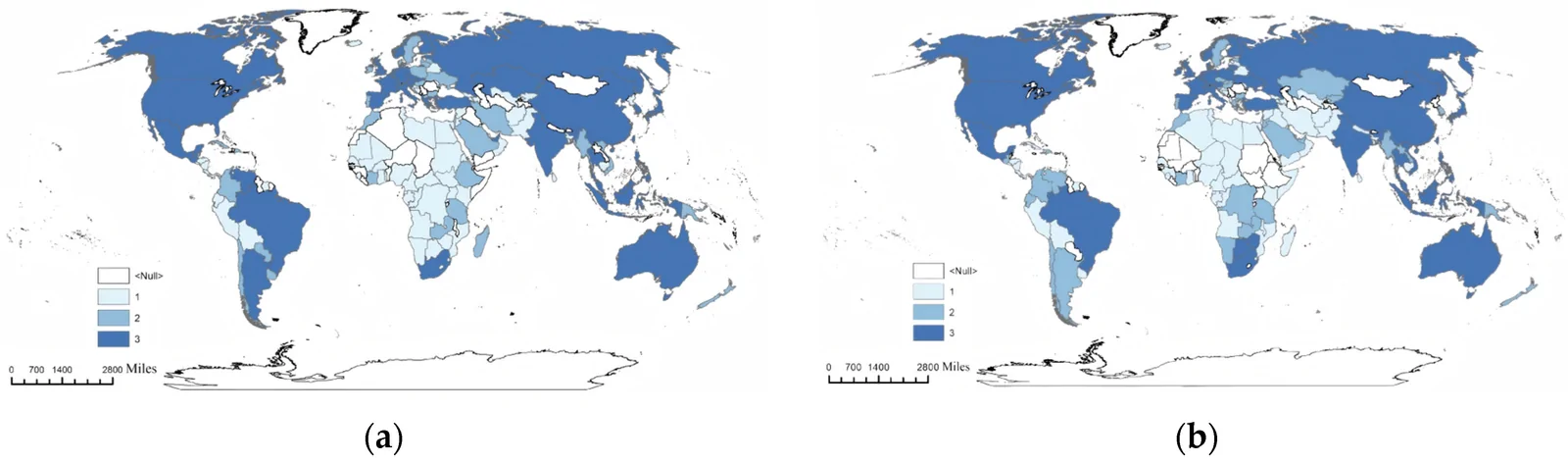
Geographic distribution map of node activity levels. Note: Figure (**a**) shows node activity levels in 2020, and Figure (**b**) shows those in 2024.

As shown in Figure 3, larger nodes indicate greater node activity, meaning that a country participates in more stages and is more deeply embedded in the nickel industry chain. China, Germany, the United States, and Japan displayed high node activity in both 2020 and 2024, reflecting broad participation in resource supply, processing, and end-use applications. Countries with high activity also possessed stronger cross-stage connectivity and therefore had greater potential influence on the multilayer network.

Between 2020 and 2024, node activity in Ukraine and Vietnam increased from M=2 to M=3, indicating that their trade links expanded from partial industrial stages to all three layers. In contrast, node activity in South Korea, the United Arab Emirates, and the Czech Republic decreased from M=3 to M=2, indicating a narrower range of participation. Overall, a small group of integrated economies maintained cross-stage connectivity, whereas most countries specialized in particular stages. This pattern demonstrates pronounced layer differentiation and specialization in the global nickel trade system.

**Figure 4:**
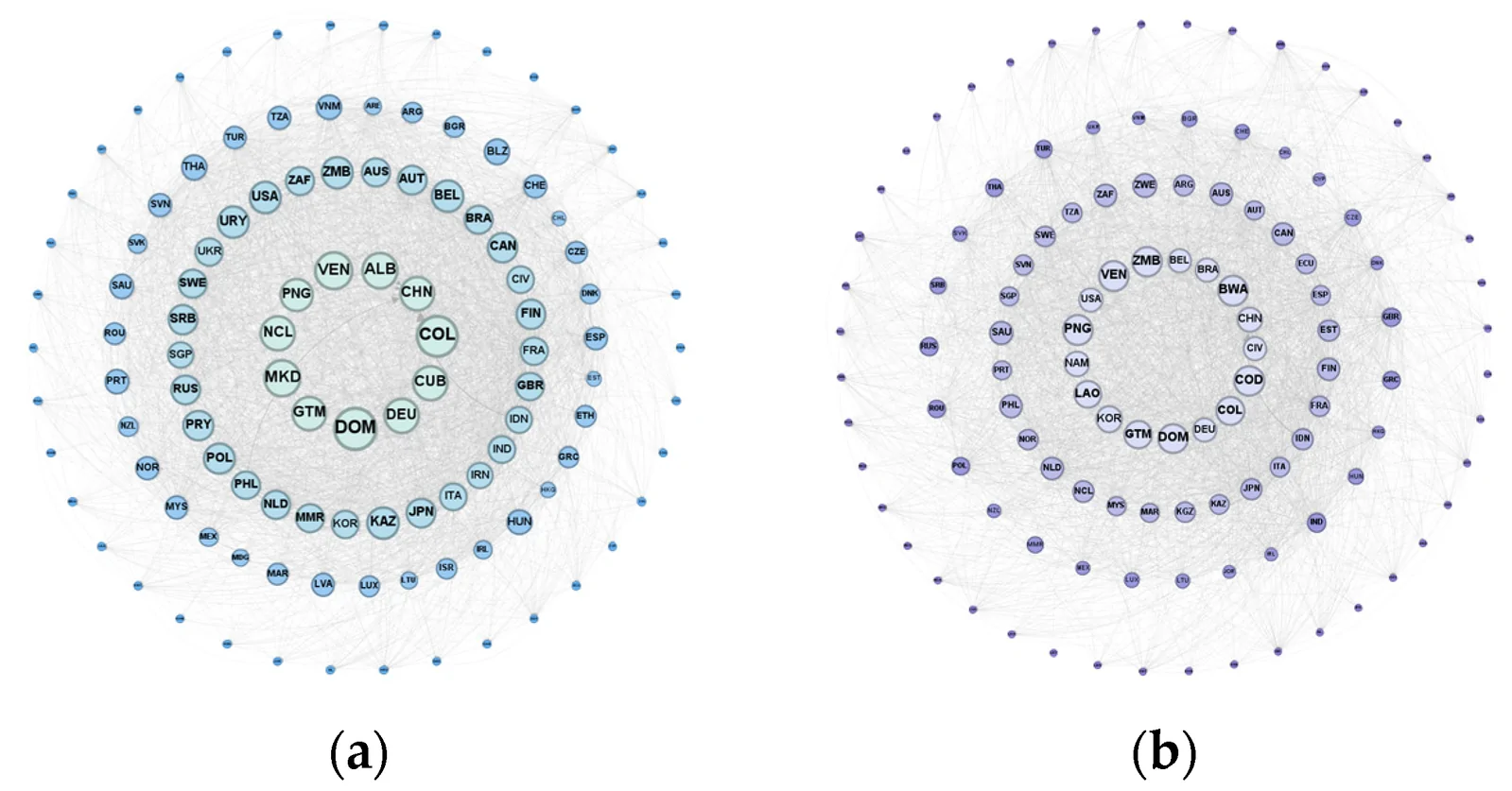
Multilayer participation coefficients in 2020 and 2024. Note: Where (**a**) represents the multi-layer participation coefficient for 2020, and (**b**) represents the multi-layer participation coefficient for 2024.

Larger nodes indicate higher multilayer participation coefficients and a more balanced distribution of trade links across the three layers (Figure 4). In 2020, Germany, China, Belgium, the United States, Canada, and Finland had relatively high coefficients, identifying them as broadly embedded economies with both extensive trade connections and sustained participation across multiple stages. These countries remained highly ranked in 2024, indicating that the cross-layer connectivity and network-support roles of the principal economies were relatively stable.

### 3.2. Supply-Risk Propagation in the Multilayer Nickel Network

#### 3.2.1. Identification of Critical Risk-Source Countries

Risk-source rankings are obtained by ranking countries according to their cascade avalanche sizes at a given effective shock-to-threshold ratio, κ. A country with a larger avalanche size receives a higher ranking. To identify critical nodes, the ten highest-ranked upstream, midstream, and downstream sources were extracted for 2020 and 2024 at different values of the effective shock-to-threshold ratio, κ (Table 3, Table 4 and Table 5).

Key risk sources in the upstream nickel trade network fell into two categories: resource-supplying countries and international trade hubs. In 2020, the Philippines and New Caledonia dominated resource-related risk, while South Korea, Germany, Japan, and the United States transmitted shocks through industrial and trading connections. The resulting structure combined supply shocks originating in resource countries with diffusion through industrial economies. The upstream network consequently remained dependent on established mining regions in Oceania and Southeast Asia, while industrial economies in East Asia, Europe, and North America acted as hubs for raw-material flows and processing trade.

By 2024, the composition of critical upstream sources had changed substantially. The Philippines and New Caledonia retained central supply-risk positions, while Guatemala, Vietnam, Indonesia, and Brazil rose in the rankings and traditional industrial economies such as South Korea, Germany, and Japan declined. This shift was consistent with the expansion of electric vehicles and power batteries, which increased nickel demand and encouraged the development of mining and smelting capacity in Southeast Asia and Latin America. Indonesia’s ore-export restrictions and domestic smelting expansion further strengthened the control exercised by resource-rich countries over upstream supply. The center of upstream risk thus shifted from industrial consumers and trade hubs toward emerging suppliers with strong resource endowments and policy leverage.

Upstream supply shocks propagated through trade links to midstream smelting and primary processing, creating cascading risks. In 2020, the midstream pattern combined resource-processing countries with trade hubs. Brazil and the Dominican Republic generated relatively large effects under low-intensity scenarios, while New Caledonia, South Africa, and Greece became more prominent as shock intensity increased. The Netherlands, Belgium, and Germany served as hubs through their established trade networks and processing capacity.

By 2024, the midstream risk landscape had been restructured. Indonesia ranked first under every shock scenario and became the most important transmission node in the global midstream network. Russia, Finland, and Cuba rose in the rankings as producers of refined nickel and high-grade nickel intermediates, while New Caledonia and South Africa declined. This transition reflected the concentration of smelting capacity in countries that integrate resource extraction with processing, particularly the transformation of midstream supply through Indonesia’s export restrictions and domestic capacity expansion. Demand for high-nickel battery materials further amplified shocks originating in major intermediate-product producers. The center of midstream risk therefore shifted from geographically dispersed smelters and European trade hubs toward integrated production centers led by Indonesia.

Supply shocks in the upstream and midstream layers propagated along the value chain to downstream processing and end-use applications. The ranking of downstream sources therefore reflected the geography of global nickel consumption and advanced manufacturing. In 2020, China ranked first among downstream risk sources under various effective shock–threshold ratios κ. As the world’s largest nickel consumer and a major producer of stainless steel and battery materials, fluctuations in its downstream industries generated the strongest network-wide effects. The United States, Finland, Belgium, Germany, and Japan also ranked highly because of their technological and scale advantages in nickel-based alloys, precision electroplating, and end-product manufacturing. South Africa, the United Kingdom, and South Korea occupied important positions through regional trade-hub functions and specialized production capacity. Downstream risk was consequently concentrated in major manufacturing and consuming economies in East Asia, North America, and Western Europe.

China remained first under every shock scenario in 2024, consistent with the rapid expansion of its power-battery industry and sustained stainless-steel production. The downstream ranking nevertheless became more diverse: Türkiye, Spain, and Greece entered the top ten, while Indonesia continued to rise as downstream capacity expanded toward resource-producing regions and emerging markets. Finland, Belgium, Germany, and Japan retained substantial transmission capacity because of technological barriers in advanced nickel materials. The downstream network therefore remained dominated by a small group of core economies but included an increasingly diverse set of secondary participants.

#### 3.2.2. Layer-Specific Responses to Cascading Propagation

**Figure 5:**
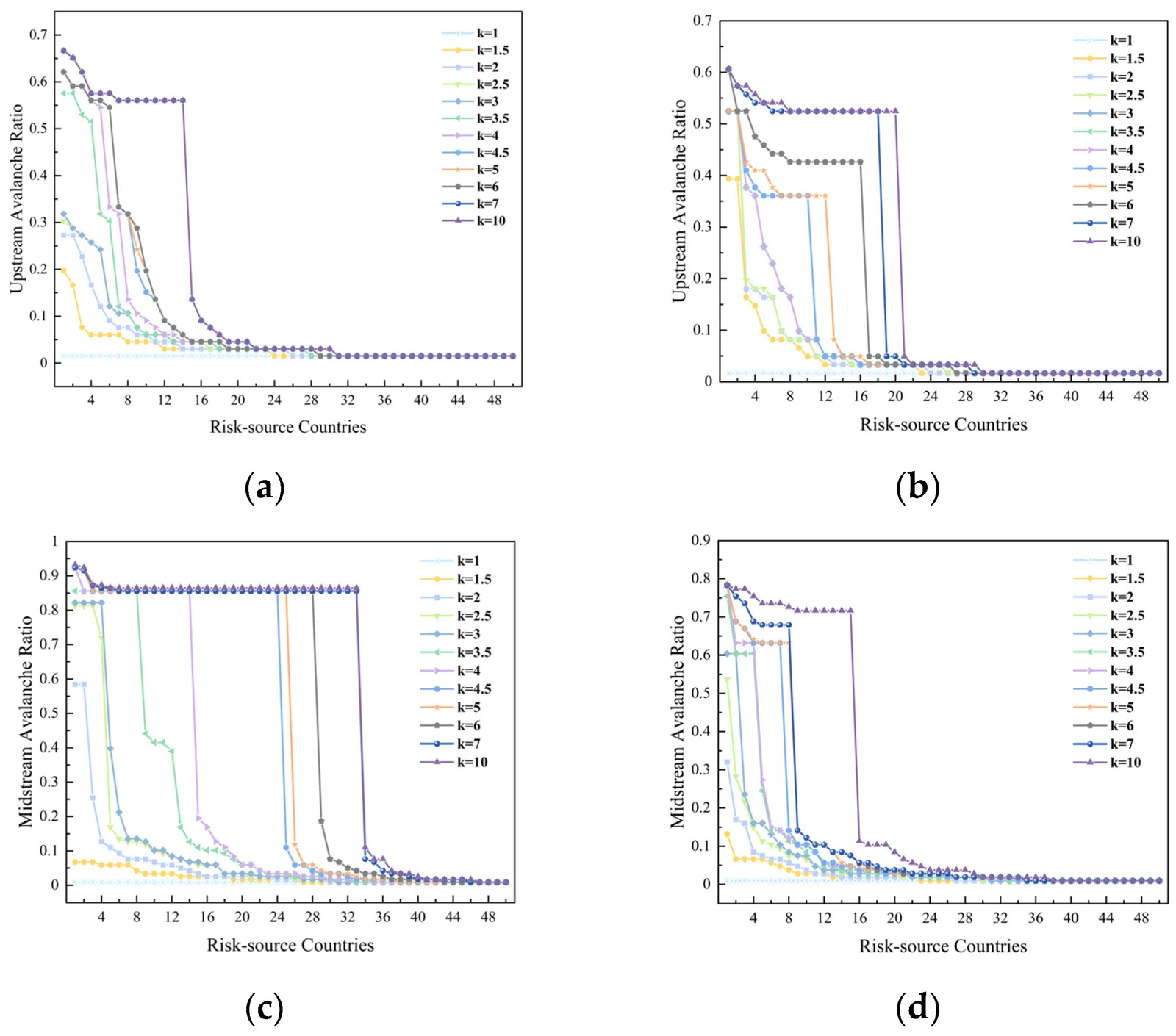

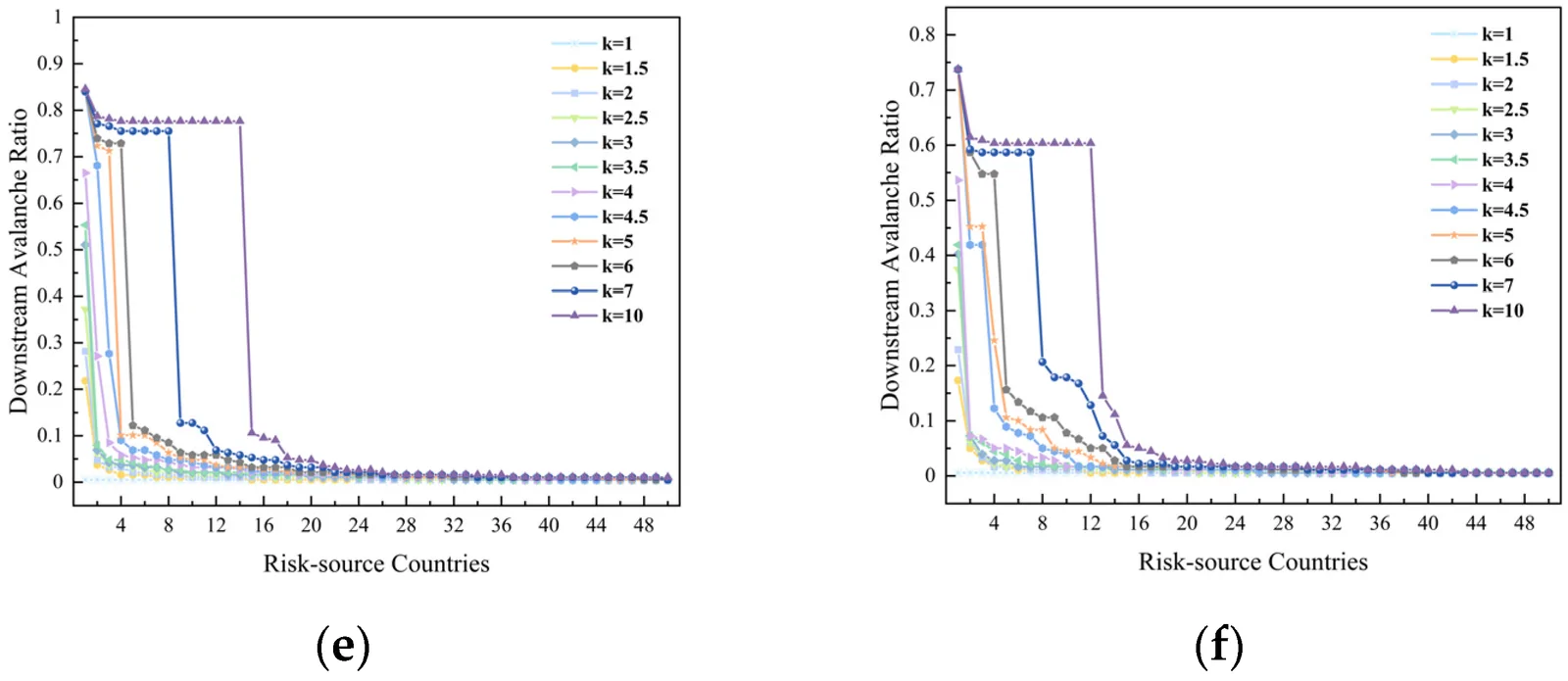
Avalanche ratios in the upstream, midstream, and downstream layers, 2020 and 2024. Note: (**a**,**b**) show the avalanche ratios in the upstream section for 2020 and 2024; (**c**,**d**) present those for the midstream section; while (**e**,**f**) display the values for the downstream section.

The changes in risk transmission avalanche ratios across the upstream, midstream, and downstream segments of the nickel resource trade network under different effective shock-to-threshold ratios are shown in Figure 5. The effective shock-to-threshold ratio was positively associated with the avalanche ratio: the higher the value of κ, the wider the scope of cascading failures induced by supply disturbances and the stronger the systemic supply risk. Taking the upstream network as an example, when κ equals 10, supply crises occurring in the top 14 risk-source countries can all trigger an avalanche ratio exceeding 56%; in contrast, when κ equals 5, only shocks from the top-ranked six countries are likely to produce cascading effects covering more than half of the nodes. A comparison of 2020 and 2024 reveals that fewer countries generated high avalanche ratios in 2024, indicating that transmission capacity had become more concentrated in leading core nodes and that the systemic influence of peripheral countries had weakened. Cascading effects were strongest in the midstream layer. In 2020, under a strong shock scenario with an κ value of 10, the 33 highest-ranked midstream sources each generated an avalanche ratio close to 90%, confirming that smelting and primary processing formed the principal amplification layer.

The nickel trade network was dominated by a small number of critical nodes. Core countries carried most trade flows and transmission capacity, whereas shocks originating from peripheral countries produced limited network-wide effects. Across the upstream, midstream, and downstream layers, the three highest-ranked risk-source countries can each trigger an avalanche ratio exceeding 60% when κ>3, indicating that supply disruptions originating from core nodes can have substantial system-wide impacts. In contrast, countries ranked below 15th generally produce high avalanche ratios only under the extreme scenario of κ=10, and have limited effects on the overall network under low- and moderate-intensity shocks.

From 2020 to 2024, the number of nodes, connectivity, and weighted trade volume increased as the industry expanded. More diverse trade paths strengthened robustness to random shocks to some extent. Once a shock exceeded the critical threshold, however, failures still cascaded through the three layers and caused widespread supply losses. Transmission capacity also became more concentrated in a small number of nodes. The resulting structure was relatively robust to random shocks but highly vulnerable to disruptions at critical nodes, particularly major resource suppliers, smelting and processing centers, and end-use consumption hubs.

#### 3.2.3. Propagation Magnitudes of Representative Countries

**Figure 6:**
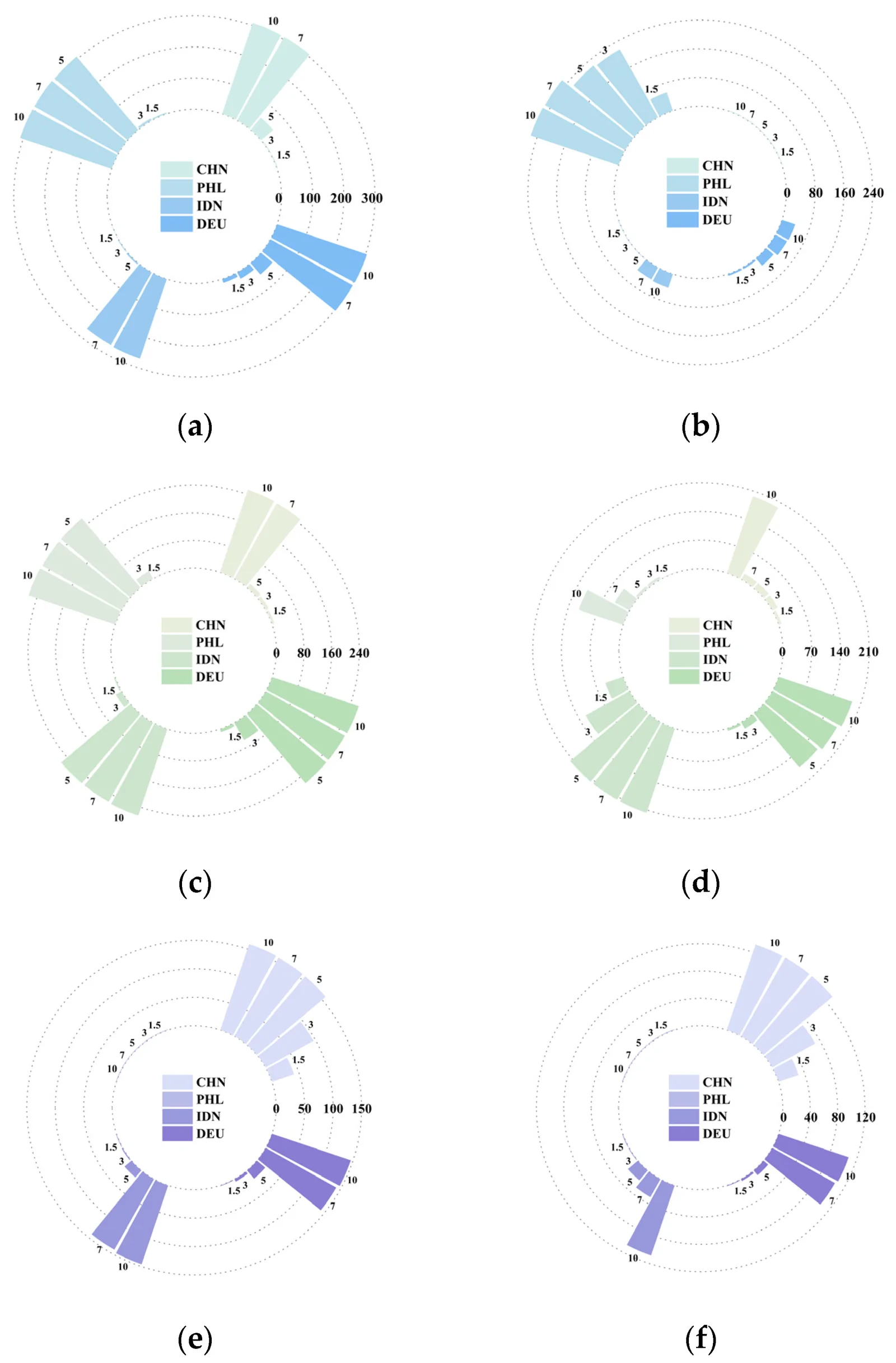
Avalanche sizes generated by China, the Philippines, Indonesia, and Germany across the three layers. Note: (**a**,**b**) show the upstream avalanche scales in China, the Philippines, Indonesia, and Germany; (**c**,**d**) depict the midstream avalanche scales across these four countries; while (**e**,**f**) illustrate the downstream avalanche scales for all four nations. The numbers on top of the bars represent κ=1.5, 3, 5, 7, 10, and the values on the dashed lines denote the number of countries.

Figure 6 presents the cascade avalanche sizes triggered by four critical countries, namely China, the Philippines, Indonesia, and Germany, in 2020 and 2024 at κ=1.5, 3, 5, 7, 10. It illustrates differences in propagation intensity and temporal evolution among the principal risk sources across the upstream, midstream, and downstream layers of the nickel industry chain. The cascade avalanche sizes associated with all four countries generally increase with κ, indicating that stronger shocks are more likely to exceed network resilience thresholds and induce cross-layer propagation. The dominant risk-source countries differ markedly across the three layers, revealing a clear functional division of roles within the industry chain.

In 2020, upstream transmission was driven jointly by resource suppliers and trade-processing hubs, including the Philippines, Germany, and Indonesia. Germany, Indonesia, and China generated substantial cascading effects in the midstream layer, indicating that smelting and processing were sensitive to disruptions originating from different types of economies. China clearly dominated the downstream layer and generated large avalanches under all shock scenarios. Its position as a global center of nickel consumption, advanced processing, and end-product manufacturing therefore gave it the strongest downstream transmission capacity.

By 2024, the transmission pattern had changed structurally. The avalanche size generated by the Philippines increased markedly in the upstream layer, confirming its growing supply weight and network influence in nickel-ore trade. Germany’s upstream role weakened as traditional industrial trading economies became less dominant at the resource stage. Indonesia’s midstream transmission capacity increased substantially following the rapid expansion of its smelting and intermediate-product industries. China remained the dominant downstream node and generated larger avalanches than the other representative countries, indicating that end-use consumption and advanced processing risks remained highly concentrated in China.

Overall, the center of risk transmission shifted from traditional industrial trading economies toward resource suppliers and countries integrating extraction with processing between 2020 and 2024. Upstream risk became more concentrated in the Philippines, midstream risk increased in Indonesia, and downstream consumption remained dominated by China. Thus, although trade routes became more diverse, systemic risks at critical stages continued to depend heavily on a few core countries.

#### 3.2.4. Propagation Pathways of Representative Countries

To further investigate risk-propagation pathways in the mineral trade network, we analyzed the paths originating from four core countries, namely the Philippines, Germany, China, and Indonesia, in 2020 at an effective shock-to-threshold ratio of κ=10. Their pathways were classified into four types: multi-round intermediary propagation, spatially clustered propagation, direct propagation, and cross-layer propagation along the industry chain.

(1) The Philippines: Multi-Round Intermediary Propagation

In multi-round intermediary propagation, the initial source first affects a small number of critical intermediary countries, which then transmits the disruption more widely. A shock originating in the Philippines first reached China, Germany, Indonesia, India, the United States, and the Netherlands. Propagation through these highly connected intermediaries subsequently extended into the midstream and downstream layers and ultimately generated cascading failures across the industry chain. The Philippine pathway therefore demonstrates how critical intermediaries can progressively amplify an initial resource-supply shock.

(2) Germany: Spatially Clustered Propagation

Spatially clustered propagation begins within a geographic region or regional production network before spreading beyond that cluster. A shock originating in Germany first affected numerous European countries, including Belgium, Switzerland, the Czech Republic, Denmark, Spain, and Finland. Risk accumulated within the European manufacturing and trade network before crossing regional boundaries and reaching other regions and layers. The German pathway therefore exhibited clear regional clustering followed by outward diffusion.

(3) China: Direct Propagation

**Figure 7:**
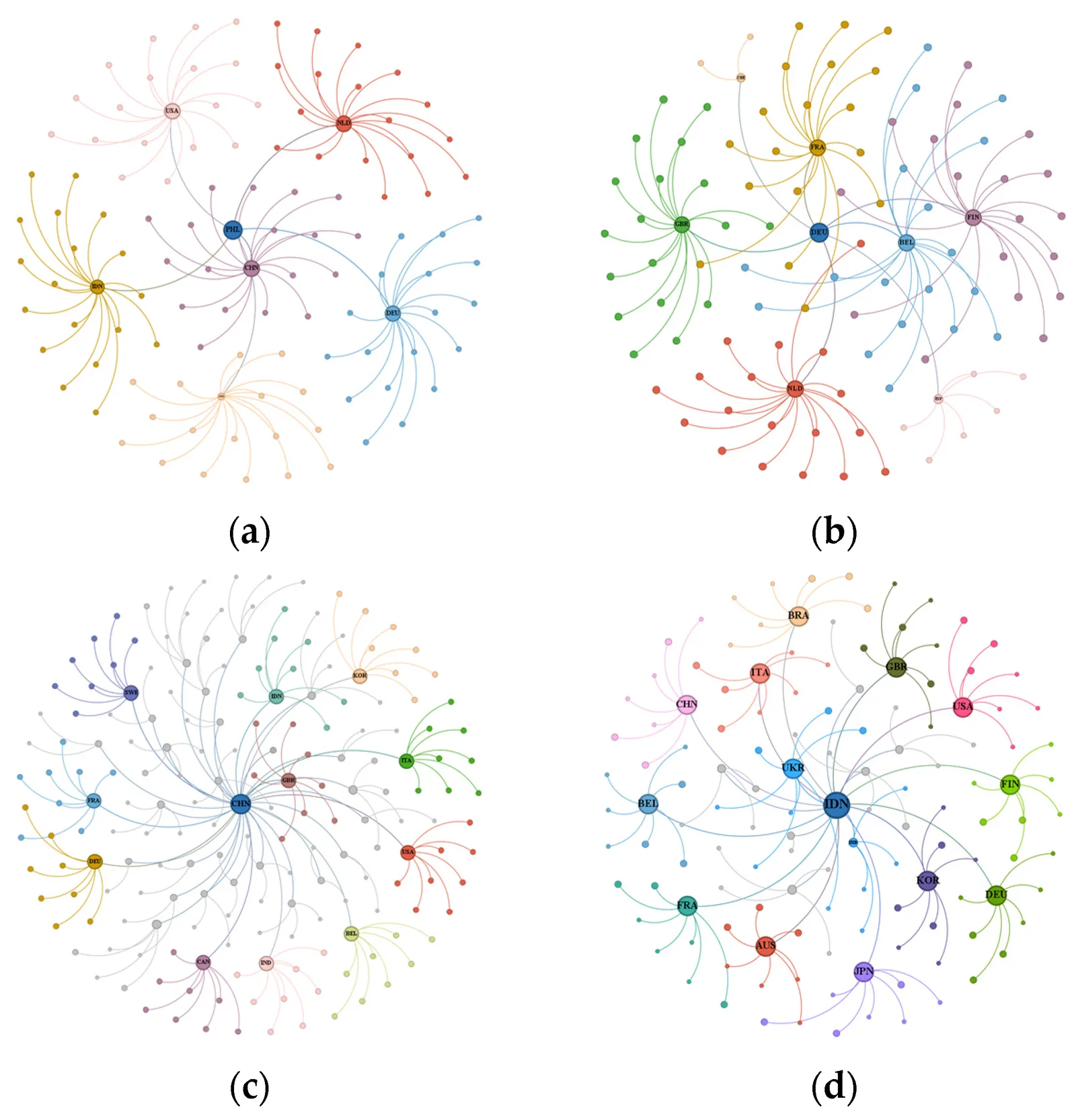
Propagation pathways originating from China, the Philippines, Indonesia, and Germany in 2020. Note: (**a**–**d**) respectively illustrate the risk transmission pathways of the Philippines, Germany, China, and Indonesia.

Direct propagation occurs when a highly influential source affects many nodes without relying on a distinct intermediary-amplification stage. Because China generated relatively limited upstream and midstream effects, Figure 7 focuses on its role as a downstream source. A shock originating in China directly affected Germany, Indonesia, the Philippines, the United States, the Netherlands, South Korea, India, and numerous other downstream economies. The disruption therefore spread rapidly and extensively through end-use and downstream-processing networks.

(4) Indonesia: Cross-Layer Propagation along the Industry Chain

Cross-layer propagation develops sequentially across production stages and is amplified as it moves between layers. When Indonesia was treated as an upstream source, the initial effect was limited to Ukraine and India. After the disruption entered the midstream layer, however, the number of affected countries increased markedly and included China, the United States, South Korea, Japan, Australia, and Finland. The Indonesian pathway therefore illustrates how a localized upstream shock can be amplified in smelting and processing before propagating to downstream consumption and end markets.

#### 3.2.5. Robustness Test

To examine the robustness of the model results to the setting of interlayer conversion coefficients, and considering that processing losses may vary across countries and product categories, this study further conducts a sensitivity analysis using alternative conversion coefficients. Specifically, the upstream-to-midstream interlayer conversion coefficient $\lambda_{12}$ is set to 0.90, and the midstream-to-downstream interlayer conversion coefficient $\lambda_{23}$ is set to 0.85. The critical risk-source countries in 2020 and 2024, together with their upstream, midstream, and downstream avalanche ratios, are then re-identified.

**Figure 8:**
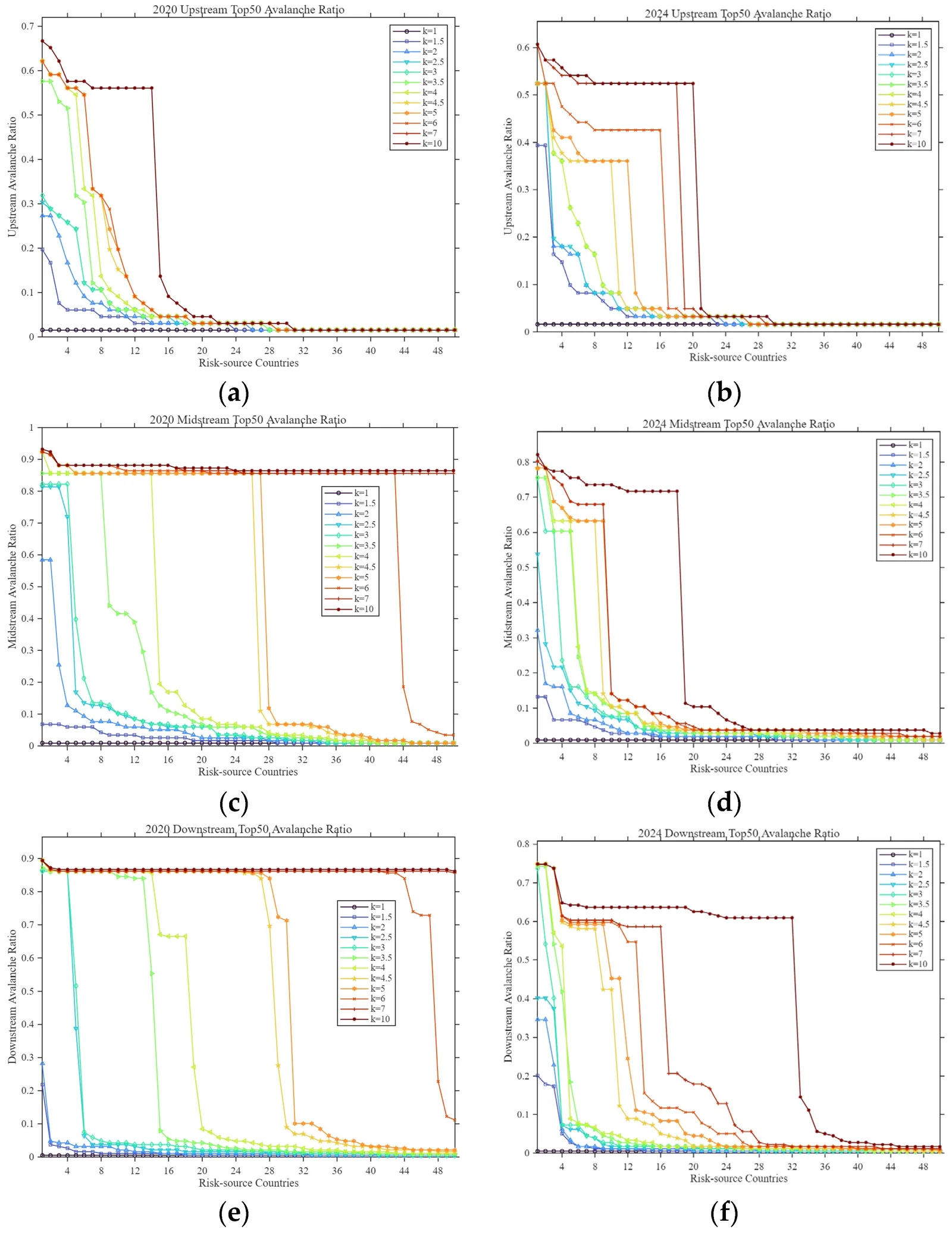
The avalanche ratios of upstream, midstream and downstream layers under robustness tests in 2020 and 2024. Note: (**a**,**b**) show the avalanche ratios in the upstream section for 2020 and 2024; (**c**,**d**) present those for the midstream section; while (**e**,**f**) display the values for the downstream section.

The sensitivity analysis results indicate that the ranking of countries identified as key risk-sources is completely consistent with the baseline scenario; the variations in the avalanche ratio across different shock coefficients are illustrated in Figure 8. After adjusting the inter-layer conversion coefficient, the specific values for the avalanche scale and avalanche proportion undergo certain changes; however, the identification results for key risk source countries, the hierarchical amplification characteristics, and the primary risk propagation patterns remain largely consistent with the baseline results, demonstrating that the main conclusions exhibit strong robustness to variations in the inter-layer conversion parameters.

## 4. Discussion

The global nickel industry chain exhibits pronounced layer-specific heterogeneity. The upstream network is relatively sparse because it is strongly constrained by resource endowments and export policies, whereas the downstream network contains more participants and broader connections, resulting in greater network accessibility. In the midstream network, fewer connections coexist with increasing trade intensity, indicating that smelting and primary processing capacity is becoming concentrated in a limited number of core countries and trade routes. The higher avalanche ratios observed in the midstream layer further demonstrate that greater connectivity efficiency does not necessarily improve supply resilience. When large trade flows depend on a small number of processing centers, local supply disruptions can propagate within the affected layer and forward to adjacent downstream layers through material-conversion relationships. The midstream layer therefore constitutes a major amplifier of supply-side cascading risk.

The strong correspondence among node centrality, the multilayer participation coefficient, and risk-propagation capacity further reveals the heterogeneous nature of the nickel trade network. Countries such as China, Germany, the United States, Belgium, Canada, the Netherlands, and Japan generally combine large trade volumes, high centrality, and extensive cross-layer connections. They therefore function as both structural hubs and potential risk amplifiers. These macro-level findings can further support micro-level supplier selection and supply-chain reconfiguration decisions under uncertainty by helping firms and policymakers identify relatively safer sourcing partners and critical vulnerability nodes. Multilayer participation alone does not necessarily increase systemic vulnerability. However, when extensive cross-layer participation coincides with high trade intensity, high betweenness centrality, and low substitutability, forward cross-layer connections can facilitate the cascading propagation of supply losses. The marked variation in avalanche ratios also suggests that the network is relatively robust to random disruptions but highly vulnerable to failures of a small number of critical nodes. Between 2020 and 2024, the center of risk shifted from traditional industrial and trading economies, including Germany, Japan, and the United States, towards resource-supplying and vertically integrated economies such as the Philippines and Indonesia. Global nickel supply security is therefore increasingly shaped by resource availability and the geographical concentration of resource control and processing capacity.

Compared with single-layer trade-network approaches, the multilayer framework captures structural differences and forward linkages across upstream resources, midstream processing, and downstream products. Combining node activity, the multilayer participation coefficient, and cascading-failure simulations also clarifies the relationships among structural position, cross-layer embeddedness, and forward supply-risk propagation. The model should therefore be interpreted primarily as a structural risk-identification and scenario-simulation framework. It captures the network outcomes formed under the combined influence of resource endowments, industrial policies, capacity expansion, trade adjustments, and changing market demand, but it does not endogenously model the economic processes that generate these changes. Thus, the results are best understood as revealing structural exposure, critical risk-source countries, layer-specific amplification, and potential propagation pathways under the observed trade configuration, rather than as forecasts of future network evolution or direct explanations of individual economic decision-making processes.

Nevertheless, the current interlayer mechanism mainly represents supply-side transmission from the upstream layer to the midstream layer and subsequently to the downstream layer. The cascading-failure model is intentionally simplified and follows a threshold-based propagation rule. It does not explicitly incorporate trade rerouting, substitution of intermediate products, time dynamics, inventory buffers, strategic reserves, price feedbacks, reverse interlayer transmission, or firm-level adaptive responses. Therefore, extreme avalanche ratios should not be interpreted as direct predictions of actual market collapse or realized supply losses. Rather, they indicate the potential extent of structural exposure embedded in the multilayer trade network under a specified shock intensity and threshold rule. The model is primarily based on trade volumes and nickel-content equivalents and does not fully account for capacity utilization, long-term contracts, product substitutability, or heterogeneous national risk tolerance and response capacity. Future research could incorporate country-specific thresholds, firm-level supply relationships, inventory dynamics, demand-side responses, trade rerouting, substitution mechanisms, price-feedback effects, and bidirectional coupling mechanisms to improve the behavioral realism of supply-chain risk simulations. Longer time series and multiple disruption scenarios would also support a more precise assessment of how policy changes, geopolitical conflicts, and growing demand from clean-energy industries reshape risk propagation across the global nickel industry chain.

## 5. Conclusions

Based on complex network theory, a multi-layered trade network for the global nickel resource industry chain was constructed. Using the cascade failure model, supply risk propagation patterns were simulated across all countries for the years 2020 and 2024. The main conclusions are as follows: (1) The network exhibited pronounced layer heterogeneity, and the midstream layer was the principal amplifier of cascading failure risks. The average degree and clustering coefficient of the downstream network were larger, while its average path length was shorter, reflecting broader market coverage and higher accessibility. The upstream network was sparse and strongly constrained by resource endowments and export policies. In the midstream layer, declining average degree and rising average weighted degree indicated a shift from growth in the number of connections toward the reinforcement of core channels. Correspondingly, the midstream layer generated larger avalanche ratios than the upstream and downstream layers under comparable shocks. (2) High-centrality countries with broad cross-layer participation largely coincided with the strongest risk-transmission nodes. China, Germany, the United States, Belgium, Canada, the Netherlands, and Japan combined high strength, betweenness, or PageRank centrality with extensive cross-layer embeddedness. Large trade volumes, participation in multiple stages, balanced cross-layer links, positions along critical routes, and limited substitutability increased the likelihood that shocks at these nodes would become systemic. (3) A small number of countries controlled most trade flows and dominated transmission. The network was therefore relatively robust to random shocks but highly vulnerable to disruptions at critical nodes. (4) The center of risk shifted from traditional industrial and trading economies toward resource suppliers and countries integrating extraction with processing. Between 2020 and 2024, upstream influence increased in the Philippines, midstream influence increased in Indonesia, and China retained its dominant downstream role. Global nickel security has consequently become less a question of aggregate resource availability than of excessive concentration in critical resources and processing capacity.

The findings support four policy implications:

First, dependence on a small number of resource suppliers, processing centers, and trade routes should be reduced by diversifying ore sources, intermediate-product supplies, and smelting capacity. Policy shifts, supply shortages, or external shocks originating from a few core countries or critical routes can trigger chain reactions across the network. Broader import portfolios, alternative supply channels, and stronger regional specialization and cooperation would increase redundancy, substitutability, and recovery capacity.

Second, countries combining high centrality, broad cross-layer participation, and strong transmission capacity, including the Philippines, Indonesia, China, and Germany, should receive priority in monitoring systems. Export policies, mining quotas, industrial policies, social instability, geopolitical conflicts, and other disruptions in these countries can propagate rapidly beyond domestic trade. A cross-layer early-warning system should therefore integrate routine monitoring, scenario analysis, and risk classification across the upstream, midstream, and downstream layers.

Third, resilience should be strengthened in midstream smelting, refining, and primary processing. Concentration of intermediate-product supply in a few countries or routes can amplify local disturbances and accelerate propagation in both directions along the chain. Diversifying processing capacity, upgrading technology, coordinating smelting across regions, and improving the circulation of raw materials and intermediate products would reduce single-node failure risk and increase the midstream layer’s capacity to absorb shocks.

Fourth, strategic stockpiles, secondary-resource recovery, and international supply-chain cooperation should be integrated into a coordinated risk-mitigation system. National stockpiles can buffer temporary interruptions; recycling and reuse can reduce dependence on primary mineral imports; and long-term agreements, regional processing partnerships, overseas joint development, and multilateral cooperation can strengthen institutional resilience. Together, these measures would limit the propagation of shocks from critical nodes and reduce the probability of industry-wide cascading failures.

## Figures and Tables

**Table 1:** Product coverage and nickel-content coefficients for the nickel industry chain.

Industrial Chain	Product Name	HS Code	Product Nickel Content/(kg/kg)
Upstream	Nickel ore	2604	0.015
Midstream	Nickel sulfate	283324	0.224
	ferronickel	720260	0.200
	High nickel matte	750110	0.750
	Mixed hydroxide precipitate (MHP)/mixed sulfide precipitate (MSP)	750120	0.600
	Refined nickel	750210	0.995
Downstream	Nickel alloy	750220	0.500
		750512	
		750522	
		750620	
		750712	
	Nickel material	750400	0.995
		750511	
		750521	
		750610	
		750711	
		750720	0.500
	Stainless steel	7218	0.058
		7219	0.040
	Battery	850730	0.091
	Scrap	7503	0.500

**Table 2:** Centrality rankings in the nickel industry-chain trade network, 2020 and 2024.

Centrality	Top 5 Strength Centrality		Top 5 Betweenness Centrality		Top 5 PageRank Centrality	
	2020	2024	2020	2024	2020	2024
Upstream	China	Philippines	Germany	China	Germany	Netherlands
	Philippines	China	China	USA	Canada	China
	New Caledonia	Indonesia	USA	Netherlands	China	Canada
	Indonesia	New Caledonia	Finland	Canada	Singapore	Brazil
	South Korea	South Korea	Canada	South Africa	Finland	Finland
Midstream	China	China	China	China	China	China
	Indonesia	Indonesia	Germany	Germany	Netherlands	Germany
	Papua New Guinea	Japan	Belgium	Belgium	India	Netherlands
	New Caledonia	Papua New Guinea	India	USA	Germany	Belgium
	Japan	New Caledonia	Netherlands	India	UK	Italy
Downstream	China	China	China	Germany	UK	Germany
	Germany	Indonesia	UK	USA	Germany	Netherlands
	Indonesia	Germany	Germany	China	Netherlands	India
	South Korea	USA	USA	India	Malaysia	Spain
	USA	India	Netherlands	France	France	France

**Table 3:** Top ten risk-source countries in the upstream layer.

Year	Risk-Source Ranking	κ				
		3	5	6	7	10
2020	1	New Caledonia	Philippines	Philippines	New Caledonia	New Caledonia
	2	South Korea	New Caledonia	China	Philippines	Philippines
	3	Germany	China	New Caledonia	China	China
	4	Mexico	Japan	Japan	Japan	Japan
	5	USA	South Korea	South Korea	South Korea	South Korea
	6	Netherlands	Germany	Germany	Mexico	Mexico
	7	Canada	Netherlands	Mexico	Belgium	Belgium
	8	Philippines	Mexico	USA	Germany	Germany
	9	Guatemala	USA	Netherlands	Malaysia	Malaysia
	10	Finland	South Africa	South Africa	Netherlands	Netherlands
2024	1	Philippines	Philippines	Philippines	Philippines	Philippines
	2	Italy	Italy	Italy	Mexico	New Caledonia
	3	USA	USA	USA	Thailand	Guatemala
	4	Belgium	Vietnam	Mexico	Indonesia	Mexico
	5	Netherlands	Brazil	Thailand	Vietnam	Thailand
	6	South Africa	Canada	Belgium	Australia	Indonesia
	7	Canada	Belgium	Vietnam	Belgium	Japan
	8	Finland	Australia	Australia	Brazil	Vietnam
	9	Guatemala	Germany	Brazil	Canada	Australia
	10	Australia	Finland	Canada	Germany	Belgium

**Table 4:** Top ten risk-source countries in the midstream layer.

Year	Risk-Source Ranking	κ				
		3	5	6	7	10
2020	1	Brazil	New Caledonia	New Caledonia	New Caledonia	New Caledonia
	2	the Dominican Republic	South Africa	South Africa	South Africa	South Africa
	3	Netherlands	Australia	Greece	Greece	Greece
	4	USA	Austria	Malaysia	Malaysia	Ukraine
	5	Germany	Belgium	Ukraine	Ukraine	Australia
	6	Austria	Brazil	Australia	Australia	Austria
	7	Philippines	Colombia	Austria	Austria	Belgium
	8	Japan	Germany	Belgium	Belgium	Brazil
	9	Russia	the Dominican Republic	Brazil	Brazil	Colombia
	10	Indonesia	UK	Colombia	Colombia	Germany
2024	1	Indonesia	Indonesia	Indonesia	Indonesia	Indonesia
	2	Belgium	Russia	Russia	Russia	China
	3	Netherlands	Finland	Finland	Finland	Russia
	4	Brazil	Cuba	Cuba	Cuba	Finland
	5	Germany	Brazil	Belgium	Belgium	Australia
	6	China	Germany	Brazil	Brazil	Colombia
	7	Russia	Netherlands	Germany	Germany	New Caledonia
	8	Finland	Belgium	Netherlands	Netherlands	Cuba
	9	USA	China	USA	USA	Belgium
	10	Colombia	Colombia	New Caledonia	Philippines	Brazil

**Table 5:** Top ten risk-source countries in the downstream layer.

Year	Risk-Source Ranking	κ				
		3	5	6	7	10
2020	1	China	China	China	China	China
	2	USA	Finland	Finland	Japan	Finland
	3	Finland	Belgium	Belgium	Finland	Japan
	4	Belgium	Germany	Germany	Belgium	Belgium
	5	South Africa	South Korea	USA	Germany	Canada
	6	UK	USA	South Korea	France	Germany
	7	Netherlands	Indonesia	UK	UK	France
	8	Germany	UK	Indonesia	Indonesia	UK
	9	France	South Africa	Türkiye	Canada	Indonesia
	10	South Korea	Italy	France	USA	India
2024	1	China	China	China	China	China
	2	USA	Belgium	Finland	Finland	Japan
	3	South Africa	France	Belgium	Belgium	Finland
	4	Belgium	Finland	France	Germany	Belgium
	5	Türkiye	Indonesia	Indonesia	France	Canada
	6	Germany	USA	Italy	UK	Germany
	7	Spain	Italy	South Korea	Italy	France
	8	Greece	South Korea	Canada	Japan	UK
	9	Italy	Germany	USA	Canada	Indonesia
	10	Japan	Türkiye	Germany	USA	Italy

## Data Availability

All data in this study were sourced from the United Nations Data Trade Database.
